# Cycle-Induced Interfacial Degradation and Transition-Metal Cross-Over in LiNi_0.8_Mn_0.1_Co_0.1_O_2_–Graphite Cells

**DOI:** 10.1021/acs.chemmater.1c02722

**Published:** 2022-02-18

**Authors:** Erik Björklund, Chao Xu, Wesley M. Dose, Christopher G. Sole, Pardeep K. Thakur, Tien-Lin Lee, Michael F. L. De Volder, Clare P. Grey, Robert S. Weatherup

**Affiliations:** † Department of Materials, University of Oxford, Parks Road, Oxford OX1 3PH, U.K.; ‡ The Faraday Institution, Quad One, Harwell Science and Innovation Campus, Didcot OX11 0RA, U.K.; § Department of Chemistry, University of Cambridge, Lensfield Road, Cambridge CB2 1EW, U.K.; ∥ Department of Engineering, University of Cambridge, 17 Charles Babbage Road, Cambridge CB3 0FS, U.K.; ⊥ Diamond Light Source, Harwell Science and Innovation Campus, Fermi Avenue, Didcot OX11 0DE, U.K.

## Abstract

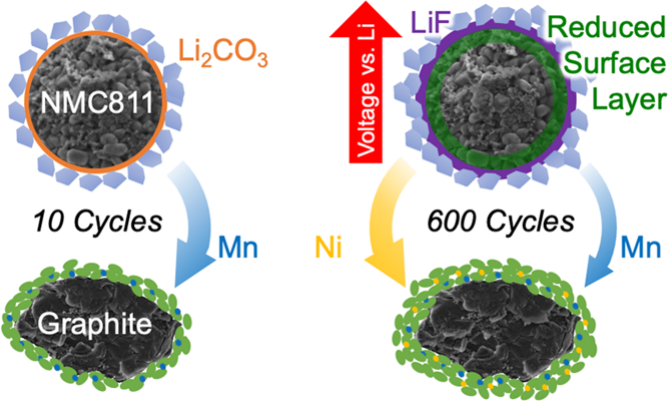

Ni-rich lithium nickel manganese cobalt (NMC) oxide cathode materials promise Li-ion batteries with increased energy density and lower cost. However, higher Ni content is accompanied by accelerated degradation and thus poor cycle lifetime, with the underlying mechanisms and their relative contributions still poorly understood. Here, we combine electrochemical analysis with surface-sensitive X-ray photoelectron and absorption spectroscopies to observe the interfacial degradation occurring in LiNi_0.8_Mn_0.1_Co_0.1_O_2_–graphite full cells over hundreds of cycles between fixed cell voltages (2.5–4.2 V). Capacity losses during the first ∼200 cycles are primarily attributable to a loss of active lithium through electrolyte reduction on the graphite anode, seen as thickening of the solid-electrolyte interphase (SEI). As a result, the cathode reaches ever-higher potentials at the end of charge, and with further cycling, a regime is entered where losses in accessible NMC capacity begin to limit cycle life. This is accompanied by accelerated transition-metal reduction at the NMC surface, thickening of the cathode electrolyte interphase, decomposition of residual lithium carbonate, and increased cell impedance. Transition-metal dissolution is also detected through increased incorporation into and thickening of the SEI, with Mn found to be initially most prevalent, while the proportion of Ni increases with cycling. The observed evolution of anode and cathode surface layers improves our understanding of the interconnected nature of the degradation occurring at each electrode and the impact on capacity retention, informing efforts to achieve a longer cycle lifetime in Ni-rich NMCs.

## Introduction

Lithium-ion batteries (LIBs) are a key technology in enabling the transition of energy infrastructure from a reliance on fossil fuels toward increasing use of intermittent, renewable energy sources.^1,2^ Despite their commercialization more than three decades ago and widespread use in portable electronics and electric vehicles, the demand for higher energy densities and increased cycle lifetimes continues.^3^ The energy densities of LIBs are largely limited by the cathode active materials, and thus, many efforts have focused on replacing the LiCoO_2_ (practical capacity 155 mAh g^–1^) used in first-generation LIBs with higher-capacity layered transition-metal oxides where some portion of the Co is exchanged for other metals. Promising among these are the LiNi*_x_*Mn*_y_*Co*_z_*O_2_ (NMC) materials, which maintain the same layered rhombohedral structure and have been widely adopted in the automotive sector. Ni cations are the main site for redox activity in NMC materials, and thus, increasing the Ni content delivers higher capacities as well as lowering the content of Co, which is both expensive and has ethical concerns over its extraction. This has led to great interest in Ni-rich NMC materials such as NMC811, where *x* = 0.8 and *y* = *z* = 0.1, which can achieve capacities of 200 mAh g^–1^ at relatively high average discharge voltages of ∼3.8 V vs Li/Li^+^.^3,4^

Unfortunately, increasing the Ni content of NMC is accompanied by increased reactivity toward the electrolyte and diminished cycle lifetime.^3,5,6^ The extent of this degradation is found to be more severe at higher potentials, making the upper cutoff potential an important factor for cycling these materials.^7^ Not only is the electrolyte increasingly oxidized at higher potentials but the NMC surface can also react and undergo phase transformations to form spinel and/or rock-salt layers and evolve gas.^8−11^ Such gas evolution can originate from the lattice oxygen, with NMC811 exhibiting a lower onset potential than NMC622 and NMC111.^12^ It can also arise from surface impurities such as Li_2_CO_3_;^12,13^ these can grow due to atmospheric impurities during material storage and typically form to a larger extent on the surface of more Ni-rich NMCs. Furthermore, transition-metal dissolution from the cathode active material has also been implicated in the capacity fading observed when NMCs and many other commercial positive electrode materials are used, particularly at high potentials and temperatures.^14−16^ Understanding the extent to which these interfacial degradation processes contribute to capacity loss, as well as the chemical mechanisms involved, is critical to increasing the cycle life of cells through the design of cycling protocols and development of effective solutions for mitigating degradation (e.g., electrode coatings, electrolyte additives).

Three main types of capacity loss can be distinguished from these degradation processes: (i) direct loss of active material can occur through the dissolution, transformation, or detachment of the particles of active material, or their effective isolation from the electrode.^17^ (ii) Slippage occurs where there is an imbalance in the rate of faradaic side reactions that proceed at each electrode, meaning their potential profiles slip with respect to each other such that their full capacity is no longer accessible.^18−20^ (iii) Impedance reduces the accessible electrode capacity at a given rate due to limited ion and/or electron transport.^21^ It is important to note that a particular degradation mechanism may contribute to more than one type of capacity loss simultaneously or at different stages of cycling. For example, the transformation of the NMC surface to a spinel/rock-salt layer may lead to the direct loss of active material through the material transformation itself and constraints it places on delithiation of the bulk NMC.^11^ It can also lead to an increased interfacial impedance and thus loss of accessible capacity due to the lower Li-ion mobility of this surface layer.^22^ Similarly, transition-metal dissolution from the cathode can potentially contribute to the direct loss of active material from the cathode, as well as migration of the dissolved transition-metal species to the graphite anode, where they are incorporated into the solid-electrolyte interphase (SEI) promoting further side reactions and thus slippage.^14^ Interestingly, the different transition metals have been shown to affect SEI stability differently, with Mn expected to be more detrimental than Ni.^23^ For Ni-rich NMC materials, the dissolution and incorporation of Ni into the SEI might be expected to take place to a larger extent than for Mn,^3^ suggesting that the lower Mn content of Ni-rich electrodes may be beneficial in this respect.^23^ However, this is yet to be satisfactorily verified, and we note that this may still be outweighed by degradation related to the increased reactivity of Ni-rich NMC surfaces.

The variety of potential mechanisms that can contribute to each type of capacity loss makes it challenging to determine how degradation proceeds, especially as the balance of different mechanisms can change over the cell’s lifetime. In this context, consideration of the interactions between different cell components is important, with half-cell studies potentially only giving a partial picture and excluding mechanisms involving cross-over between the cathode and anode.^24,25^ Furthermore, the inclusion of a lithium counter/reference electrode, or other electrode materials for that matter, can inadvertently introduce new degradation mechanisms or alter existing mechanisms.^26^ Studies that involve accelerated stress tests (e.g., elevated potentials, temperatures) can similarly introduce new mechanisms or emphasize certain mechanisms above others. This motivates the study of full cells under moderate cycling conditions to establish a more complete picture of cell degradation that remains relevant to commercial applications of Ni-rich NMC materials.

Herein, we investigate NMC811 vs graphite full cells with additive-free carbonate electrolytes, which were cycled for various cycle numbers (up to 1000) and then characterized using ex situ surface-sensitive X-ray spectroscopies. Using full-cell upper cutoff voltages of 4.2 V vs graphite at room temperature, we focus on degradation occurring during more realistic extended battery cycling rather than arising from accelerated degradation related to stress tests at high voltages or temperatures. Electrochemical impedance spectroscopy (EIS) and differential voltage analysis (DVA) combined with synchrotron-based X-ray photoelectron spectroscopy (XPS) and near-edge X-ray absorption fine structure (NEXAFS) measurements reveal how electrochemical signatures of aging correspond to interfacial degradation occurring at the electrode–electrolyte interfaces. We consider the different chemical states of each electrode and relate this to the observed electrochemical behavior to determine how different chemical degradation processes contribute to capacity fade. We show that during the first ∼200 cycles capacity fading primarily originates from electrode slippage associated with electrolyte reduction, with the graphite SEI continuously increasing in thickness and incorporating transition metals dissolved from the cathode, particularly Mn. During further cycling, capacity fade resulting from a decreasing amount of utilizable NMC material is found to be more significant. This corresponds with the NMC electrode reaching higher potentials at the end of charge as a result of anode slippage and is accompanied by reduction of the NMC surface, transition-metal dissolution, and removal of the remaining Li_2_CO_3_ species. Furthermore, the continuing incorporation of transition-metal species at the anode and thickening of the SEI is observed, with the relative proportion of Ni compared to Mn increasing with cycle number, approaching unity after ∼600 cycles. This study thus provides insight into the interfacial degradation processes occurring when Ni-rich NMCs are used in full cells, including the interplay between reactions occurring at the cathode and anode, helping to inform strategies to improve cycle life.

## Experimental Section

### Cell Assembly

NMC electrodes (provided by Argonne National Laboratory’s Cell Analysis, Modeling, and Prototyping facility) were punched into 14 mm discs containing 90% LiNi_0.8_Mn_0.1_Co_0.1_O_2_ (NMC, Targray), 5% poly(vinylidene fluoride) (PVDF, Solvay), and 5% carbon black (C45, Timcal), with a mass loading of 9.12 mg cm^–2^ and a porosity of 32.5%. Graphite electrodes (from the same source) were punched out into 15 mm discs containing 91.83 wt % graphite (Hitachi), 2 wt % carbon black (C45, Timcal), 0.17 wt % oxalic acid, and 6 wt % PVDF (Kureha), with a mass loading of 6.35 mg cm^–2^ and a porosity of 30.3%. NMC811 vs graphite coin cells (2032 size, grade 304 stainless steel) were assembled in an Ar-filled glovebox together with a Celgard 3501 separator and 50 μl LP57 (1 M LiPF_6_ in EC/EMC, 3:7 by vol., SoulBrain MI). Electrodes and coin cell parts were dried at 120 °C for >12 h under vacuum, with the Celgard separator dried at 60 °C for >12 h under vacuum.

### Electrochemical Cycling

The cells were cycled at room temperature using a galvanostatic cycling protocol on an Arbin battery cycler. Five cells underwent constant-current constant-voltage (CCCV) charging to 4.2 V followed by a constant-current discharge to 2.5 V for 12, 122, 302, 602, and 1002 cycles. Initially, two formation cycles were performed at a C-rate of 0.05C, followed by cycling at a C-rate of 0.5C (C-rate was calculated based on a practical capacity of 185 mAh g^–1^ for the NMC active material). Every 50th cycle, the C-rate was changed to 0.05C for electrochemical characterization.

### DVA Fitting

The reference 0.05C cycles from the cycling data were converted to d*V*/d*Q* profiles and curve-fitted using reference data obtained from NMC and graphite half-cells.^27^ The NMC reference cell was assembled using NMC as the positive electrode, lithium as the negative electrode, Celgard 2500 separator, and 40 μL of LP57 electrolyte. It was cycled between 2.5 and 4.5 V at a C-rate of 0.05C assuming a practical capacity of 185 mAh g^–1^ for the NMC active material. The graphite reference cell was assembled using graphite as the positive electrode, lithium as the negative electrode, Celgard 2500 separator, and 40 μL of LP57 electrolyte. It was cycled between 2.0 and 0.01 V at a C-rate of 0.05C assuming a practical capacity of 330 mAh g^–1^. These reference cells were also used in a previous study.^27^

### EIS

The cell used for EIS was cycled similarly to the other full cells with aging at a 0.5C rate but with three 0.05C reference cycles following every 100 cycles. The EIS was performed at 3.8 V after every third reference cycle using a frequency range between 10 kHz and 10 mHz and applying a 5 mV voltage amplitude on a Biologic BCS805 cycler. The EIS data have been reported in a previous study.^27^

### X-ray Spectroscopy

XPS measurements were performed at the I09 beamline of the Diamond Light Source to characterize the surface chemistry of the electrodes. The discharged cells (2.5 V) were disassembled in an Ar-filled glovebox [O_2_ < 0.1 ppm, H_2_O < 0.1 ppm], and the electrodes were rinsed with DMC. The samples were then transferred to the measurement chamber without air exposure, using an inert transfer arm sealed with a gate valve. Spectra were acquired using different photon energies for excitation, allowing information arising from different depths to be obtained. Hard X-ray photoemission spectroscopy (HAXPES) measurements were performed at excitation energies of 2.2 and 6.6 keV, whereas for soft X-ray photoemission spectroscopy (SOXPES) measurements, the excitation energy was changed between core levels to yield photoelectrons with kinetic energies of ∼315 eV. Probing depths corresponding to 95% of the elastically emitted photoelectrons were calculated according to^28^
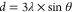
1where *d* is the probing depth, λ is the inelastic mean free path (IMFP) of the photoelectrons at the specific energy, and θ is the take-off angle, i.e., the angle between the analyzer and the sample surface (∼103° in this case). All spectra were measured at the same spot, making it possible to exclude spot-to-spot variations between the energies. Long measurements might cause beam damage, and this was checked by comparing the signal from the same F 1s or O 1s core level before and after the long measurements, and no major beam damage was observable for the nonmetals at both 2.2 and 6.6 keV. The spectra acquired for graphite electrodes were energy-referenced by setting the hydrocarbon peak to 285.0 eV, whereas the NMC spectra obtained at 2.2 and 6.6 keV were energy-referenced by setting the carbon black feature to 285.0 eV. Most of the SOXPES spectra (O 1s, F 1s, and C 1s) were energy-referenced against the carbon black/hydrocarbon peak of C 1s spectra obtained at the same excitation energy. Low binding energy core levels (P 2p and Li 1s) were calibrated against a Au 4f peak measured for a clean, gold foil at the same excitation energy, with the Au 4f energy set based on the energy difference with respect to the carbon black calibration measured at an excitation energy of 600 eV. After background subtraction, the spectra were intensity-normalized to unity, making the area equal. Atomic percentages were calculated according to^29^
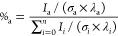
2where *I* is the intensity before the normalization, σ is the cross section, and λ is the IMFP of the photoelectrons. The inelastic mean free path was assumed to be the same as polyethylene, and the cross section was determined by piecewise cubic Hermite spline interpolation.^30,31^

The SEI and cathode-electrolyte interphase (CEI) thicknesses were calculated according to^28,32−34^
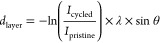
3where *d*_layer_ is the surface layer thickness, *I*_cycled_ is the area of a peak only present in the bulk from a cycled electrode (Li*_x_*C peak for SEI and lattice oxygen peak for CEI), *I*_pristine_ is the area of the corresponding peak from a pristine sample, λ is the inelastic mean free path (IMFP) of the photoelectrons in the surface layer, and θ is the take-off angle. This equation assumes that the IMFP is the same throughout the surface layer. It should also be noted that these calculations assume flat samples with homogeneous concentrations of the relevant species. This is not the case for composite electrodes; however, the calculations are still expected to provide reasonable estimates of thickness.

NEXAFS measurements were also performed at the I09 beamline of Diamond Light Source. The spectra were obtained by dividing the drain current from the sample by the incident photon flux obtained from the mirror current, giving the total electron yield (TEY). For the spectrum of the Ni L-edge after 10 cycles on the graphite electrode, which has very low intensity, additional features related to periodic storage ring top-up were removed by subtracting fitted Lorentzian functions at energies where significant changes to the ring current occurred. The spectra of the NMC electrodes were energy-calibrated with respect to the transition-metal edges of NMC electrodes from the literature.^35^ The spectra of the graphite electrodes were energy-calibrated using the same offset as for the NMC samples. The transition-metal spectra of the NMC electrodes were normalized to the peak intensity of the L_3_ peak after the removal of a straight line background. The O K-edge spectra of the NMC electrodes were background-subtracted using a straight line fitted to the pre-edge region, followed by intensity normalization to the postedge region at 550 eV. The intensities of the transition-metal peak and Li_2_CO_3_ peak were calculated after removing a straight line background beneath the corresponding peak.

Reference spectra of the transition metals were simulated by the software CTM4XAS version 5.5.^36^ In all simulations, the Slater integrals F_dd_, F_pd_, and G_pd_ were set to 1 and the spin–orbit coupling core and valence were also set to 1. Further details of the fitting parameters are seen in Table 1, where *x* indicates that the parameter was not included in the simulation.

**Table 1:** Parameters Used for Simulation of the NEXAFS Spectra

valence	symmetry	10 Dq	Δ	*U*_dd_	*U*_pd_	*T* (e_g_)	*T* (t_2g_)	color in figure
Ni^2+^	O_h_	0.8	8	6	7	2	1	blue
Ni^2+^	O_h_	0.8	1	0	0	1.5	1	gray
Co^2+^	O_h_	0.8	x	x	x	x	x	blue
Mn^2+^	O_h_	0.3	x	x	x	x	x	blue

## Results and Discussion

### Full-Cell Electrochemistry

**Figure 1:**
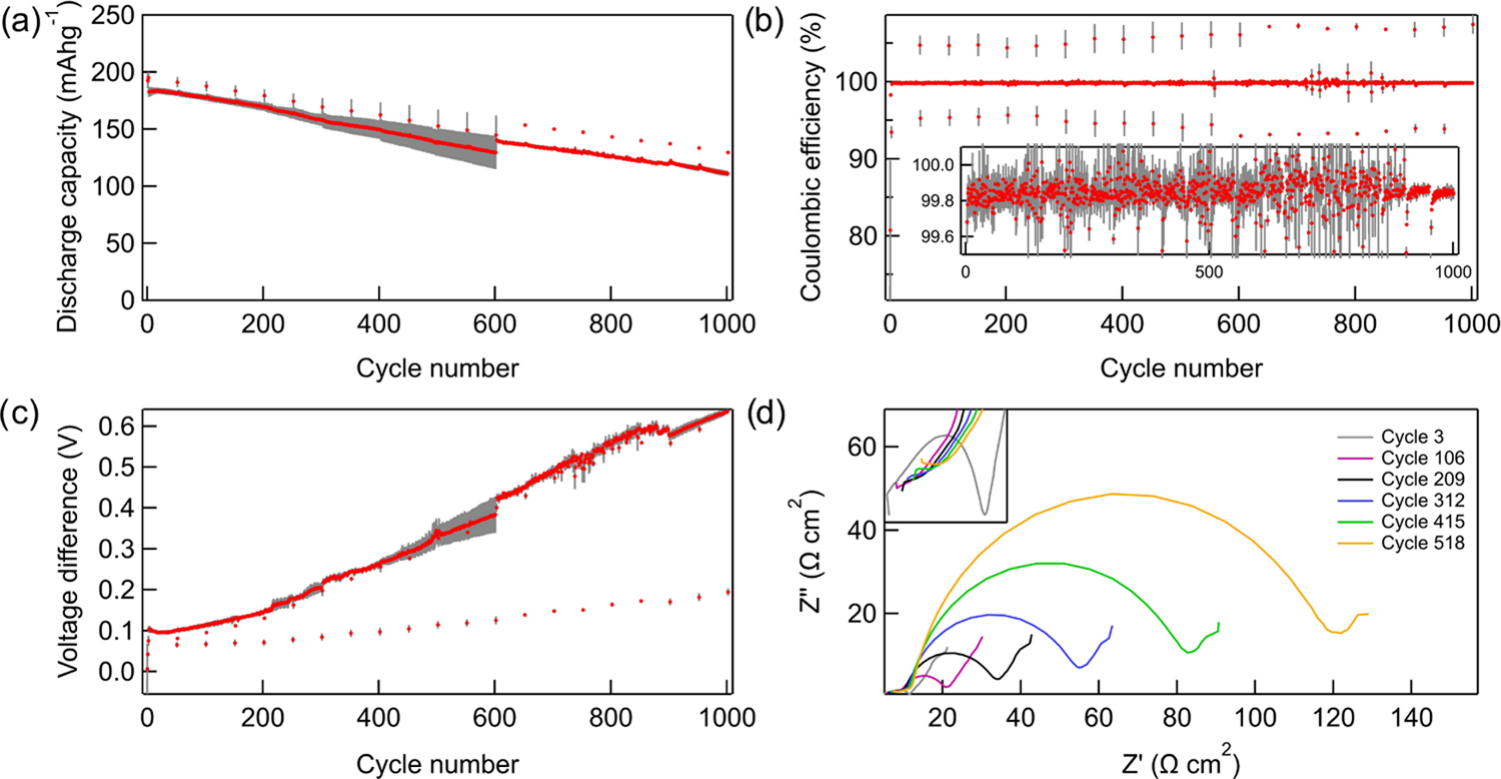
Electrochemical performance of NMC811 vs graphite cells cycled for different numbers of cycles at 0.5C, but with every 50th cycle performed at 0.05C. The gray lines correspond to 1 standard deviation of the data. (a) Discharge capacity vs cycle number. (b) Coulombic efficiency vs cycle number. (c) Voltage hysteresis between charge and discharge vs cycle number. (d) Nyquist plots obtained from EIS after different cycle numbers, performed at 3.8 V, with a 5 mV amplitude in the frequency range of 10 kHz to 10 mHz. The inset shows a magnified view showing the high-frequency semicircle from cycle 3.

Figure 1 shows the cycling performance of the NMC vs graphite cells. After 602 cycles, ∼66% of the initial capacity is retained based on the 0.05 C-rate cycling data, highlighting that without mitigating measures such as additives or known surface coatings, the capacity of the full cells decays relatively quickly. The periodic reference cycles at 0.05C show a higher capacity than the 0.5C cycles; however, their rates of fading are similar. This indicates that for the cycling conditions used, the observed rate of capacity fading is independent of overpotential, which might otherwise lead to incomplete delithiation/lithiation of the electrodes for a given cell cutoff voltage. The relative independence from overpotential is attributable to the constant potential hold at the end of charge and the low value of the lower cutoff potential of 2.5 V compared to many other studies. The constant potential hold ensures more complete delithiation of the NMC and lithiation of the graphite than what is possible from only the constant-current charging. Similarly, the low cutoff potential used during discharge (2.5 V) will also at least partly compensate for larger overpotentials on discharge. The average Coulombic efficiency for the cycles remains at ∼99.83%, excluding the initial cycle where SEI formation takes place and the reference cycles (Figure 1b). We note the cycle-to-cycle variation results from the limited accuracy of the cycling equipment, but the trend across multiple cycles is nevertheless clearly apparent. The Coulombic efficiency is somewhat lower than previous reports of >99.9% for apparently similar NMC811 vs graphite cells,^37,38^ indicating that a reasonably high proportion of charge is being lost to side reactions. The average voltage difference between charge and discharge shown in Figure 1c reveals that overpotential increases with cycle number, occurring especially rapidly for the 0.5C cycles. This is consistent with the EIS results (Figure 1d), which show a semicircle at lower frequencies that continuously increases with cycle number, attributable to an increasing charge-transfer resistance with cycle number.^39^ The EIS data here combine the contributions from both electrodes; however, previous studies on Ni-rich NMC cycled vs graphite have shown that the NMC electrode contributes most significantly to the charge-transfer resistance increase.^40^ The much smaller high-frequency semicircle coming from surface layer resistances also exhibits changes;^41^ however, the data obtained do not cover the whole semicircle, making further interpretation challenging. The electrochemical data therefore show that the cells undergo capacity fading that affects the measured capacity at both slow (0.05C) and faster (0.5C) cycling rates, while side reactions with the electrolyte continue and the cell resistance increases.

**Figure 2:**
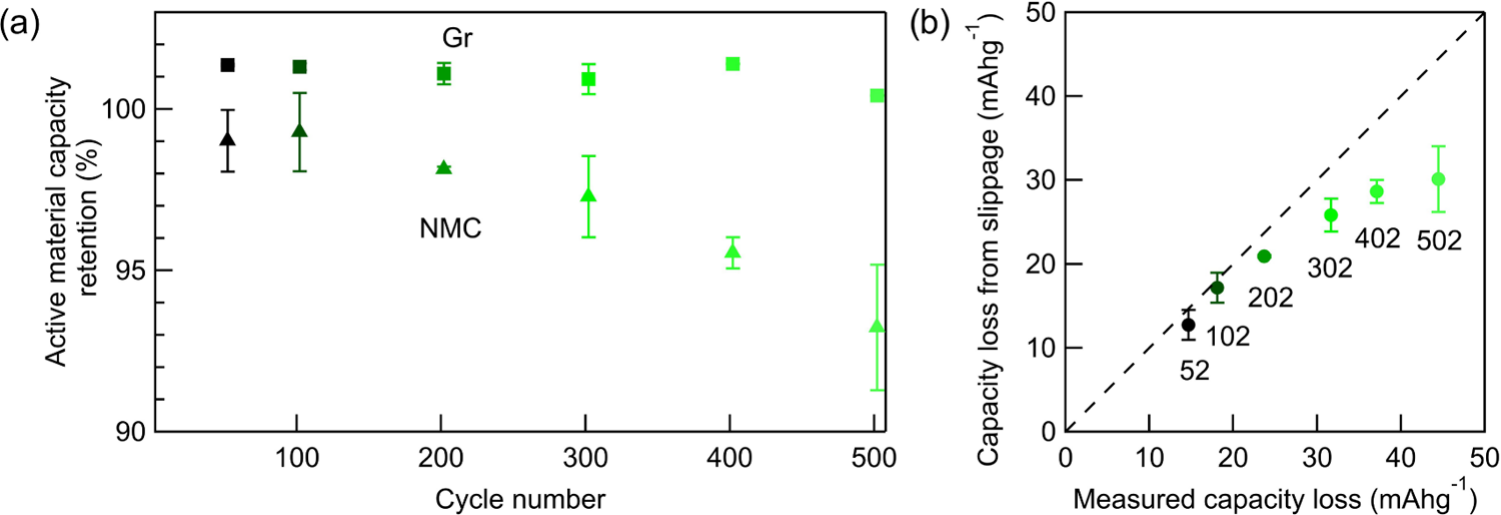
Results of differential voltage analysis (DVA) applied to the 0.05C cycles of NMC811 vs graphite cells. (a) The NMC and graphite active material capacity retention with cycle number. (b) Capacity loss due to electrode slippage compared to the measured capacity loss as a function of cycle number. The dashed line corresponds to a ratio of unity.

To better identify the processes contributing most significantly to capacity fading, DVA was applied to the 0.05C reference cycles. This involved plotting d*V*/d*Q* vs capacity and fitting half-cell data to the full-cell data, as described in ref (27). After curve fitting, it was possible to determine the extent of electrode slippage (i.e., the relative alignment of the fitted half-cell d*V*/d*Q* profiles), as well as the amount of accessible active material with cycle number, as shown in Figure 2. It is observed that the amount of graphite active material increases slightly compared to the second cycle and is relatively constant thereafter varying by <1%. The NMC, on the other hand, gradually loses active material capacity as the cycle number increases, although this loss does not affect the full-cell capacity loss in the first 200 cycles (Figure 2b). After 200 cycles, the accessible NMC active material capacity decreases more rapidly. In Figure 2b, it is seen that the measured capacity loss from the first ∼200 cycles corresponds closely to the capacity loss due to electrode slippage, i.e., the extent of reduction side reactions exceeds that of oxidation side reactions, meaning the electrode potential profiles shift with respect to each other limiting the accessible capacity. From the individual DVA plots in Figure S1, it is apparent that it is the graphite electrode limiting the discharge capacity, which corresponds to there being more reduction side reactions than oxidation side reactions occurring in the cell throughout the cycle life, consistent with prior literature.^42,43^ After ∼200 cycles, there is a growing contribution from another capacity loss process or processes (in addition to slippage), as shown by the deviation in the slope from unity. The turning point after ∼200 cycles in Figure 2a,b is attributable to additional potential stress on the cathode, brought about by the ongoing loss of active lithium causing electrode slippage and leading to an increase in the graphite potential at end of charge compared to previous cycles.^27^ An increased end-of-charge potential of the graphite, in turn, means that the NMC will be charged to a higher potential. The change in potential profile caused by slippage is schematically shown in Figure S2, where a description of the term slippage is also included. Similar trends have previously been observed where three slow reference cycles at 0.05C were used instead of one.^27^

### Cathode Characterization

**Figure 3:**
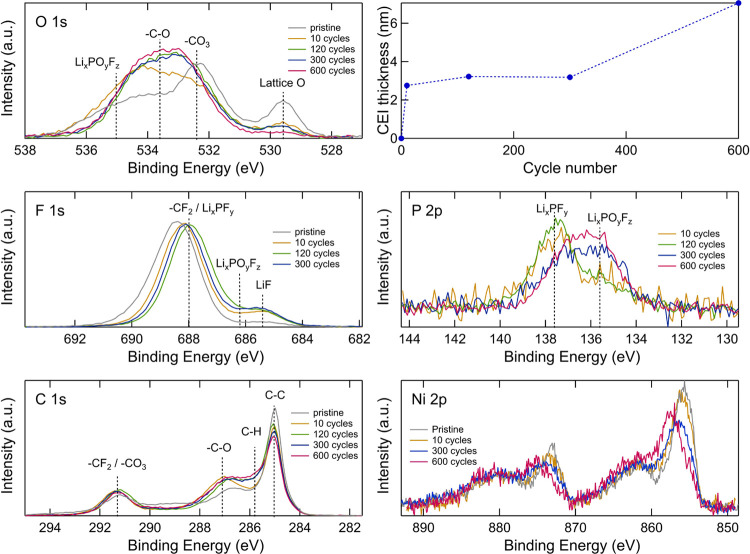
O 1s, F 1s, P 2p, C 1s, and Ni 2p core-level XP spectra of NMC electrodes after different numbers of cycles obtained with an excitation energy of 2.2 keV, and CEI thickness calculated based on the intensity of the lattice oxygen peak from the O 1s spectra.

To further elucidate the side reactions that contribute to cell degradation, the NMC electrodes were analyzed by photoelectron spectroscopy. Figure 3 shows the O 1s, F 1s, P 2p, C 1s, and Ni 2p spectra of the NMC electrodes as well as the calculated CEI thickness. The spectra were obtained using an excitation energy of 2.2 keV, which corresponds to a probing depth of ∼12 to 18 nm (depending on the core-level energy). In the O 1s region, the intensity of the lattice oxygen peak at ∼529.6 eV is seen to decrease with cycle number, indicating an increasing surface layer thickness.^44,45^ The increase of CEI thickness is quantified based on the intensity of the lattice oxygen peak in the O 1s spectra and by considering polyethylene (λ = 5.0 nm) as an approximation of the CEI chemistry. This reveals an increase from about 3 nm after 10 cycles to 7 nm after 600 cycles. It should be noted that estimated errors from similar calculations range from 7 to 45%, depending on the nature of the surface layer.^46^ For inorganic surface layers, the standard deviation in thickness was found to be about 10%, whereas a larger deviation was found for organic surface layers.^46^ The most significant change in lattice oxygen intensity occurs between the pristine and 10-cycle sample, indicating a rapid thickening during initial surface layer formation and then more gradual CEI growth during long-term cycling. A peak at ∼532.4 eV is apparent on the pristine sample corresponding to −CO_3_, likely Li_2_CO_3_.^47,48^ After cycling, increased contributions are observed at ∼533.0 and ∼534.3 eV related to electrolyte degradation and attributable to the formation of −CO_3_ and −C–O species, e.g., polyethers.^24,49^ The F 1s spectra show a species at ∼685.4 eV for all cycled samples, which is assigned to LiF formation.^50^ The intensity ratio of LiF to other fluorine species increases with cycle number, although this change is modest following the first 10 cycles, indicating its relative chemical stability with further cycling. In the P 2p spectra, on the other hand, changes take place with increasing cycle number. The peak at ∼137.6 eV corresponds to Li*_x_*PF*_y_*^51,52^ and decreases, while the peak at ∼135.6 eV that corresponds to Li*_x_*PO*_y_*F*_z_*^51,53^ increases with cycle number, consistent with the continuing reaction of the Li*_x_*PF*_y_* species or LiPF_6_ salt in the electrolyte. The decrease in Li*_x_*PF*_y_* suggests that this species is mainly formed during the initial cycling and then degrades during subsequent cycling. After the first 10 cycles, the C 1s spectra show higher intensities at binding energies of ∼285.8, ∼287.1, and ∼291.3 eV corresponding to hydrocarbons, −C–O, and −CO_3_, respectively.^44,54^ Thereafter, the peak corresponding to hydrocarbons slightly increases, whereas the peak at 285.0 eV corresponding to carbon black decreases with cycle number, indicating increasing coverage of the electrode by an organic surface layer. The Ni 2p spectra are seen to evolve with cycle number, with a shift toward higher-binding-energy peaks for samples that have been aged longer, with the most significant change apparent between 300 and 600 cycles. This suggests a change in the chemical composition of the Ni species after long-term cycling, possibly due to the formation of NiF_2_.^55^ We were, however, unable to further deconvolute the spectra to quantify the ratio of Ni-containing compounds (and furthermore to understand the surface reconstruction and spinel/rock-salt layer formation phenomenon) due to the complex line shape of the Ni 2p and the many possible overlapping contributions. The HAXPES spectra for the Mn 3p, Co 3p, and Ni 3p core levels can be seen in Figure S3. The Ni 3p evolution with cycle number again suggests a change in the Ni species. There are, however, no discernible changes in the relative intensities of the transition metals, suggesting that either transition-metal dissolution occurs following the stoichiometric ratio or that any changes in relative intensity are too small to distinguish due to the large transition-metal content in NMC.

The corresponding SOXPES spectra show that there is less variation in the degradation products present on the outer surface with cycle number (see Figure S4), although we note that differences in elemental concentrations are not taken into account. No significant differences in the O 1s and F 1s spectra were observed, while many of the differences in the carbon spectra can be attributed to increased CEI coverage of the carbon black and binder after increased cycling rather than the formation of different compounds. In the P 2p spectra, an increase in Li*_x_*PO*_y_*F*_z_* species is observed, similar to that seen for the measurements at 2.2 keV.

**Figure 4:**
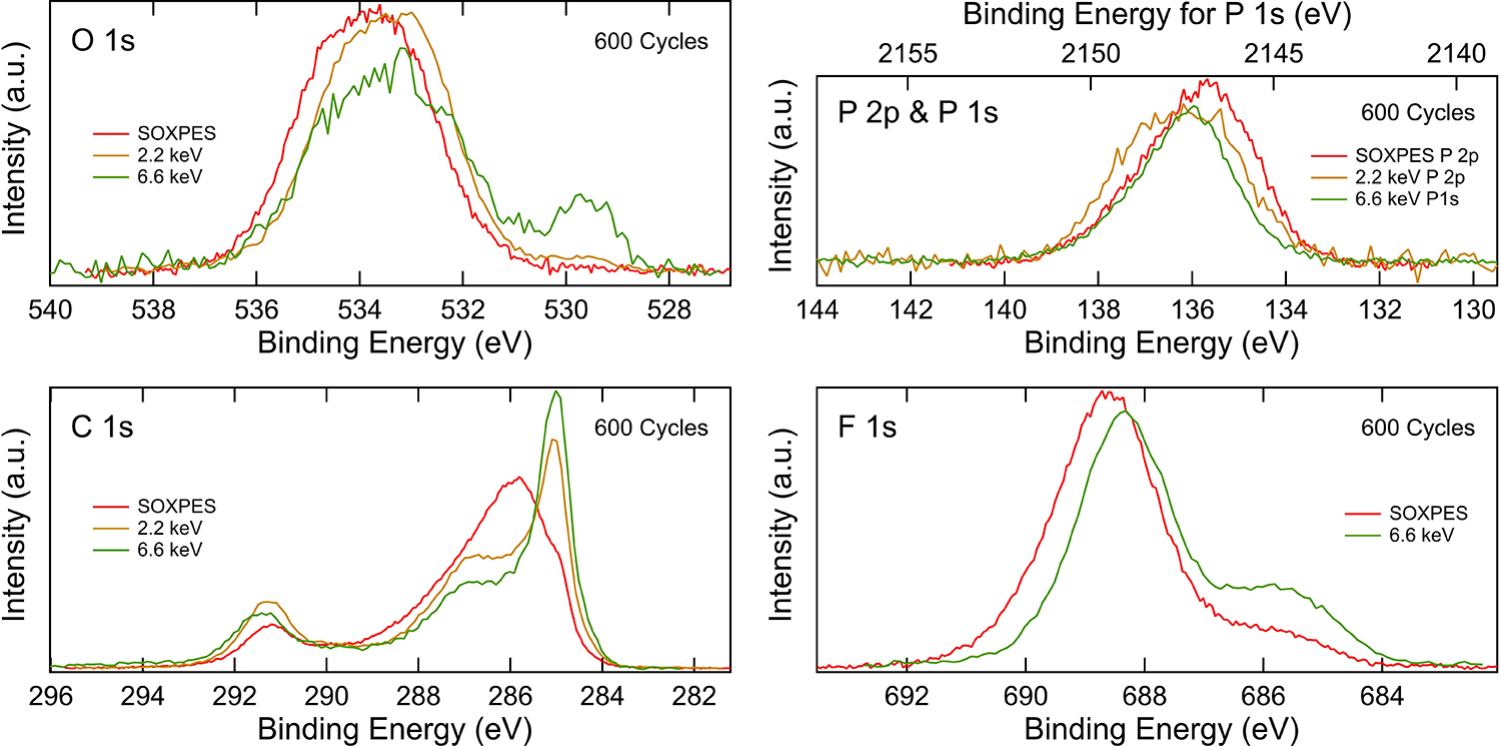
O 1s, P 2p, P 1s, C 1s, and F 1s core-level XP spectra of NMC electrodes after 600 cycles obtained with excitation energies of 2.2 keV (yellow), 6.6 keV (green), and photoelectron kinetic energies of ∼315 eV (red).

Figure 4 shows how the spectra of the 600-cycle NMC cathode change depending on excitation energy used, where the low-energy SOXPES data were measured at kinetic energies of ∼315 eV (excitation energies of 450–1000 eV) and the HAXPES data were measured at excitation energies of both 2.2 and 6.6 keV. These correspond to probing depths of ∼4, ∼12–18, and ∼41–46 nm (depending on the core-level binding energy), respectively. The O 1s spectra show that the lattice oxygen peak in the NMC at ∼529.6 eV increases for higher excitation energies, whereas the contribution from organic species at higher binding energies decreases, confirming that the organic species are formed on the surface of the NMC particles. Similarly, for the C 1s spectra, it is seen that the contribution from the carbon black at 285.0 eV increases with higher excitation energies, while the intensities from surface species, e.g., hydrocarbons and −C–O, decrease. In the F 1s spectra, the peak attributable to LiF at ∼685.4 eV increases with increasing excitation energy, showing that the LiF is mainly present in the inner part of the surface layer.^56^ The difference in the F 1s with excitation energy in Figure S6 becomes more apparent with increasing numbers of cycles, indicating that more LiF is formed in the inner surface layer during long-term cycling, making the variation in chemical composition with depth more pronounced. The P 2p spectra also show differences with excitation energy, with the peak at ∼135.6 eV more intense at lower excitation energies, indicating that more Li*_x_*PO*_y_*F*_z_* is present relative to Li*_x_*PF*_y_* in the outer part of the surface layer, while Li*_x_*PF*_y_* is more dominant deeper into the layer. Interestingly, this variation with depth is found to persist throughout cycling (Figure S7). It should be noted that given the order of measurements performed, it cannot be excluded that changes to the P 2p and P 1s spectra may, in part, arise due to beam damage.

**Figure 5:**
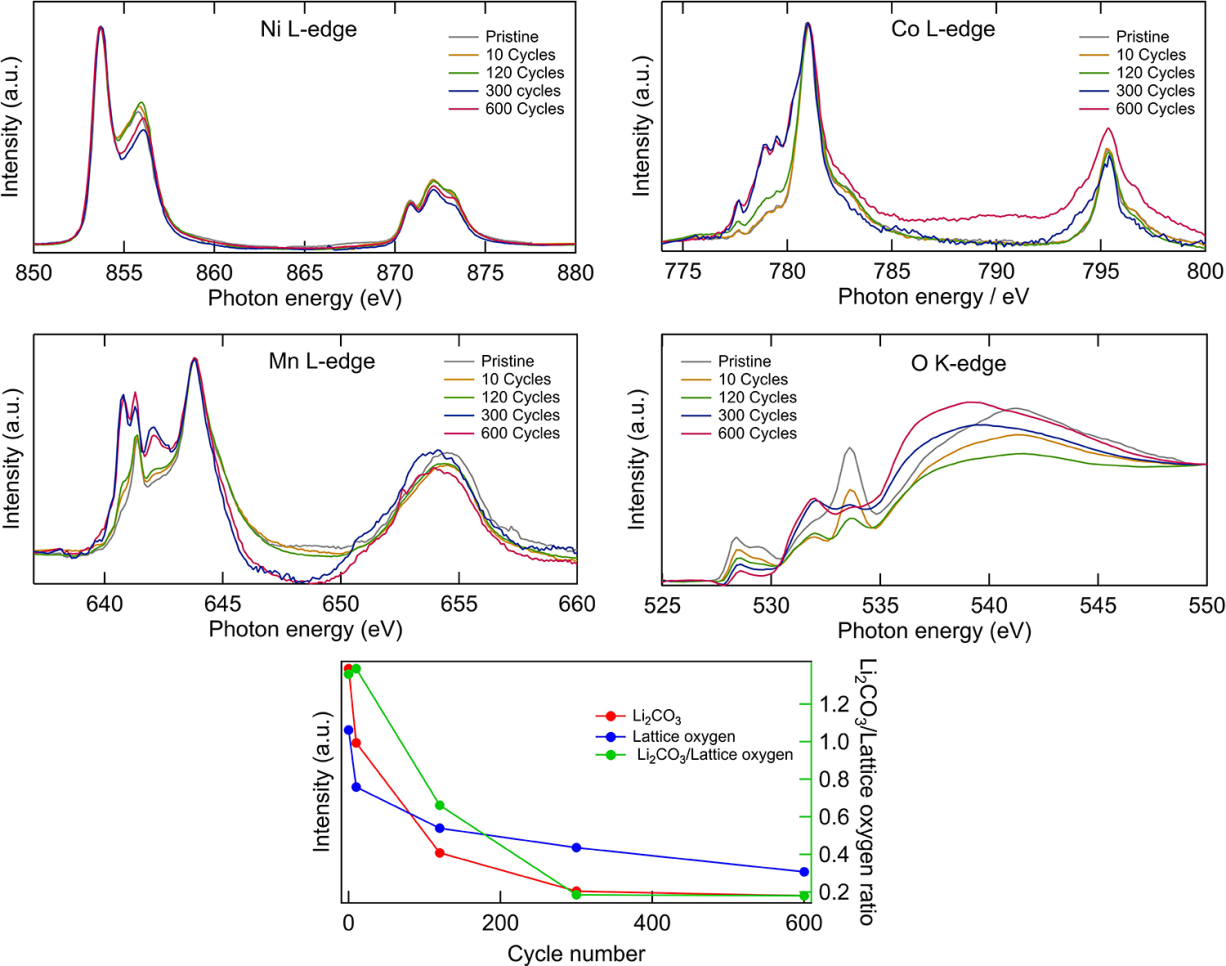
Ni, Co, Mn L-edge and O K-edge NEXAFS spectra of NMC electrodes after different numbers of cycles. The area of the Li_2_CO_3_ peak at 534.0 eV, the area of the metal oxide peaks at 528.8 and 529.8 eV (lattice oxygen), and the ratio between these peaks plotted vs the cycle number.

To reveal changes occurring on the NMC811 surface, the oxidation states of the transition metals close to the NMC surface were investigated using L-edge NEXAFS measured in TEY mode (∼10 nm probing depth).^57,58^ The spectra of the Ni L-edge (Figure 5) show features at 853.7 eV attributable to Ni^2+^ and at 855.8 eV related to both Ni^2+^ and Ni^3+^.^22,59,60^ The significant lowering in the intensity of the feature at 855.8 eV between 120 and 300 cycles indicates reduction in the Ni oxidation state, confirming the formation of reduced Ni-containing species as a result of the surface transformation.^59^ Reduction of the Co and Mn at the surface is also observed in the Co L-edge, Mn L-edge NEXAFS results (Figure 5). In the Co L-edge spectra, the main peak at 781.0 eV corresponds to Co^3+^ in the low spin state, whereas the shoulder peaks at 779.5, 778.9, and 777.7 eV correspond to Co^2+^, likely in the high spin state.^22,59,61^ With increasing cycle number, these shoulder peaks become more prominent, indicating partial reduction of the Co. The Mn L-edges show concomitant variations with cycle number. The peaks at 641.4 and 643.8 eV correspond to Mn^4+^, and the shoulder peaks at 640.8 and 642.1 eV correspond to Mn^3+^ or Mn^2+^.^62,63^ The pristine electrode shows predominantly Mn^4+^, while the 10- and 120-cycle electrodes show slightly increased Mn^3+^/Mn^2+^ contributions. After 300 cycles, the contributions from the reduced Mn have increased significantly. This emergence of reduced Ni, Mn, and Co is consistent with the NMC811 surface transforming during cycling to a spinel/rock-salt phase, as has previously been observed.^22,64,65^ Phase transformation of the NMC is expected to reduce the lithium diffusivity in the material due to the loss of its layered structure where lithium can diffuse freely.^22^ This likely contributes to the increased overpotential seen during the 0.5C cycling in Figure 1, as a lower Li diffusion rate would increase the polarization of the electrode.

In the corresponding O K-edge spectra (Figure 5), two distinct peaks are seen at 528.8 and 529.8 eV, attributable to O 1s core electron excitations into the hybridized transition-metal 3d–O 2p orbitals of the NMC.^35,59^ These lattice oxygen peaks decrease as the cycle number increases, which has previously been suggested to be due to oxygen loss from the NMC;^50^ however, the depth-resolved HAXPES measurements herein (see Figure S5) instead suggest that the thickening of the CEI layer is likely the main cause. Interestingly, the ratio between the two peaks also changes, with the high energy peak becoming less distinct with increasing cycling and almost indiscernible after 300 cycles. One potential explanation could be a difference in the SoC as a result of the electrode slippage, which has been confirmed by the differential voltage analysis discussed above. However, Yoon et al. previously showed that changes in this O K-edge feature are relatively modest (little change in shape and <10% variation in intensity) for SOC between 0 and 20% in NMC111. Considering that the SoC difference here is <15% based on the DVA, this is unlikely to yield the observed changes in relative intensity of the 528.8 and 529.8 eV components.^57^ Instead, we attribute this to changes in the composition of the NMC surface, particularly the transition-metal ratios. Similar variations have previously been observed in Co*_x_*Mn_3–*x*_O_4_ compounds as the ratio between Co and Mn changes^66^ and between different NMC compositions.^67^ The observed changes in ratio with cycle number are thus consistent with the dissolution of transition metals from the NMC surface in a nonstoichiometric ratio, although they could alternatively reflect preferential migration of certain transition metals between the NMC bulk and surface. Given that the 529.8 eV peak is attributable to Mn^4+^ and that significant Mn incorporation into the anode SEI is observed with cycling (see below), preferential dissolution of Mn appears the most likely explanation.

**Figure 6:**
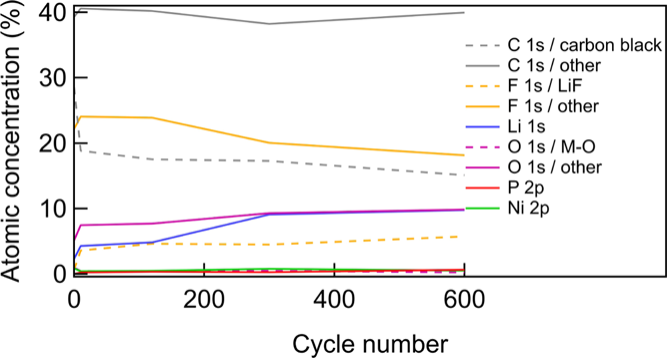
Elemental quantification of the species at the surface of NMC electrodes based on survey spectra obtained at an excitation energy of 2.2 keV, with the ratio between different carbon, fluorine, and oxygen components determined from C 1s and O 1s spectra obtained at 2.2 keV and from F 1s spectra obtained at 6.6 keV.

Alongside these changes, a peak gradually emerges at 532.2 eV, which is attributable to the formation of a reduced surface layer, consistent with the reduced oxidation states observed in the transition-metal L-edge data (i.e., Ni^2+^, Mn^2+^, Co^2+^).^68^ A peak is also observed at 534.0 eV, which is attributable to carbonate species, which most likely corresponds to Li_2_CO_3_, although partial contributions from transition-metal carbonates cannot be completely excluded.^35,59,69^ The formation of thick Li_2_CO_3_ coatings due to air exposure has previously been shown to significantly decrease the cycle life and increase the resistance of NMC and NCA electrodes, making this an important consideration for achieving long-term cycling and high C-rate performance.^13,70^ We note that the electrodes used herein have all been carefully stored in glovebox environments, but still, some Li_2_CO_3_ formation is detectable presumably resulting from residual H_2_O and CO_2_ present during storage. The Li_2_CO_3_ peak decreases in intensity with increasing cycle number, suggesting that either the Li_2_CO_3_ remains present in the inner CEI while the CEI thickens above it or that it decomposes during cycling. The intensity of the Li_2_CO_3_ peak decreases exponentially with cycle number, whereas the intensity of the lattice oxygen peaks decreases almost linearly following the first 10 cycles, showing that changes to the Li_2_CO_3_ peak cannot be fully accounted for by CEI coverage, i.e., the Li_2_CO_3_ decomposes during long-term cycling. Furthermore, the depth-resolved O 1s and C 1s spectra of Figure 4 do not suggest a discernible increase in carbonate species with depth. Previously, carbonate layers have been shown to decompose after only a few cycles, with no clear evidence for them remaining partially intact after hundreds of cycles.^71^ For NCA electrodes, it has even been reported to decompose during the first cycle.^59,72^ From the data herein, it is clear that the decomposition takes place over many cycles, even for electrodes that have not been exposed to air and thus have relatively limited carbonate layers, further highlighting the influence of surface impurities on the electrochemical performance during extended cycling. It has also been reported that the decomposition of Li_2_CO_3_ is accompanied by the formation of LiF and CO_2_ in LiPF_6_-based electrolytes, which correlates well with the LiF quantification presented below (see Figure 6).^13^ Contributions from −C–O species are expected above 535 eV,^73^ but these are hard to distinguish due to overlap with contributions from other species, in particular, the broad peaks above 535 eV attributable to hybridized states of O 2p with transition-metal 4s and higher unoccupied orbitals of the NMC.^57^

Figure 6 summarizes how the relative atomic concentrations of the elements on the surface of the NMC electrodes evolve with cycle number. The carbon black concentration shows an initially rapid decrease and continues to gradually decrease with cycle number, consistent with the CEI forming and then gradually thickening with continued cycling. Other species such as LiF and phosphorous-containing compounds increase slightly in concentration due to contributions from LiPF_6_ residue and associated decomposition products. The increase of LiF in the CEI is likely to be more pronounced than the calculated concentration suggests, as depth-resolved measurements introduced in Figure 4 show that LiF is mainly present in the inner CEI. Due to the inherent surface sensitivity of XPS, these inner CEI species will therefore contribute less to the measured photoemission signal. This growth in LiF content of the CEI correlates with the increased cell resistances observed in Figure 1, supporting the suggestion of previous studies of NMC electrodes that LiF is can be partly responsible for this.^50^ Similarly, the oxygen and lithium concentrations also increase due to the ongoing electrolyte degradation on the NMC electrodes. The reasonably constant ratios of the Ni 3p, Mn 3p, and Co 3p peaks in Figure S3 indicate that variations in the relative transition-metal concentrations are modest across the cycle numbers considered. This evolution of atomic concentrations during continued cycling is consistent with the chemical changes already discussed based on XPS, and together, these provide a picture of the CEI layer continuing to evolve during ongoing cycling, with electrode–electrolyte degradation proceeding over many cycles.

### Anode Characterization

**Figure 7:**
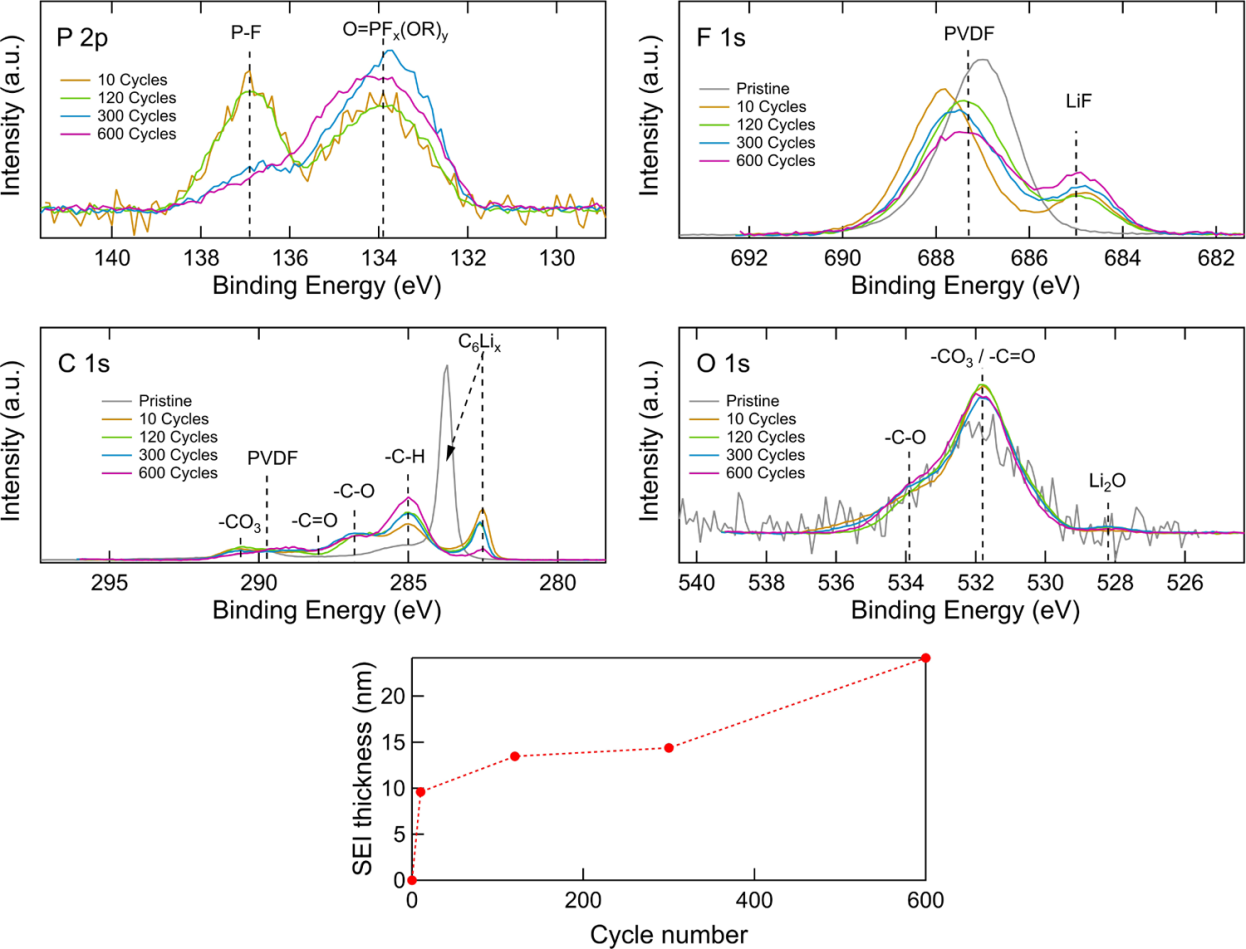
P 2p, F 1s, C 1s, and O 1s core-level XP spectra and SEI thickness calculations of graphite electrodes after different numbers of cycles, obtained with an excitation energy of 2.2 keV.

The graphite electrodes were also investigated by photoelectron spectroscopy to reveal the corresponding evolution of their surface chemistry. Figure 7 displays the P 2p, F 1s, C 1s, and O 1s core-level spectra for the graphite electrodes measured using HAXPES at an excitation energy of 2.2 keV and the SEI thicknesses calculated based on the C_6_Li*_x_* peak in the C 1s spectra. The C 1s spectra show that the relative intensity of the hydrocarbon peak at 285.0 eV increases with cycle number, indicating that hydrocarbons continue to build up within the SEI layer during prolonged cycling. The peak at 286.8 eV that corresponds to −C–O increases significantly during the initial cycles but then shows only slight increases with further cycling, indicating that it primarily relates to initial SEI formation.^74^ The −C–O may originate from a number of compounds, including esters −C(=O)–O–.^32,75^ Initially, the peak at 290.6 eV that corresponds to −CO_3_ increases after SEI formation and then gradually decreases with cycle number, likely due to further decomposition although coverage from other SEI species cannot be excluded. The F 1s spectra show a main peak at about 687.3 eV, which is primarily attributable to the PVDF binder, and its decreasing intensity with increasing cycle number is consistent with increasing SEI coverage.^76^ The peak at 685.0 eV corresponds to LiF^77^ and increases with cycle number, indicating that the contribution from LiF compared to that from the other fluorine species increases. LiF is typically assumed to be quite stable but may increase the resistance of the SEI layer,^78^ as noted earlier for the CEI. The P 2p spectra show two peaks at 136.9 and 133.9 eV corresponding to P–F and O=PF*_x_*(OR)*_y_*, respectively.^29,32^ With increasing cycle number, the O=PF*_x_*(OR)*_y_* peak increases as the P–F peak decreases, indicating the formation of more O=PF*_x_*(OR)*_y_*. The F 1s and P 2p spectra thus indicate ongoing decomposition of the LiPF_6_-based electrolyte, with the formation of various degradation products through electrochemical initiation and reactions with impurities including trace amounts of water, as is already well-established.^79−81^ The O 1s spectra show a small peak at 528.2 eV corresponding to Li_2_O and two peaks at 533.9 and 531.8 eV corresponding to −C–O and carbonates, respectively.^32,75,82^ It should be noted that the pristine sample contains only small contributions from oxygen-containing species, as apparent from the low signal-to-noise ratio.

The calculated thickening of the SEI layer with cycle number shows an increase from ∼10 nm after 10 cycles to ∼24 nm after 600 cycles. This ongoing thickening of the SEI layer during long-term cycling confirms that continuous electrolyte reduction takes place. The SEI thickness increases at a much faster rate than the CEI (∼14 nm compared to ∼4 nm between 10 and 600 cycles; see Figure 3), supporting the conclusion that electrolyte reduction at the graphite electrode is primarily responsible for the electrode slippage that leads to the majority of capacity fading observed. Although we note this does not account for species dissolved into the electrolyte or which were removed during the sample preparation.

**Figure 8:**
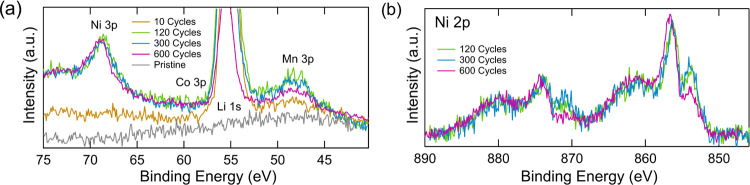
Core-level XP spectra of graphite electrodes obtained with an excitation energy of 2.2 keV. (a) Ni 3p, Co 3p, Mn 3p and Li 1s spectra. The 300- and 600-cycle samples were intensity-normalized to have the same Ni 3p intensity as the 120 cycles. (b) Ni 2p spectra.

In addition to probing core levels directly associated with electrolyte decomposition products, Ni, Co, and Mn core levels were also acquired to detect the incorporation of transition-metal species dissolved from the cathode into the SEI. We note that survey spectra do not reveal any contributions from transition metals associated with other cell components, e.g., Al, Cu from current collectors or Fe, Cr from the steel coin cell casing. The relatively small electrolyte volume used avoids cell flooding, which may account for the absence of any species related to the steel. Figure 8a shows the Ni 3p, Co 3p, Mn 3p, and Li 1s regions measured at 2.2 keV, where during extended cycling Ni and Mn are clearly seen to accumulate. To aid with comparison, all spectra are normalized to the same Ni intensity except for the pristine and 10-cycle samples that have too low Ni 3p intensities. Following initial cycling (10 cycles), a distinct Mn 3p peak is observed, whereas a clear peak from the Ni 3p region cannot be discerned. As cycling continues, the Ni 3p becomes much more prominent after 120 cycles and the relative intensity of Ni to Mn continues to increase with further cycling, showing a change in the ratio of transition-metal ions being deposited on the graphite with cycle life. The surface shows a similar concentration of Ni and Mn present after long-term cycling (see Figure 10), despite the 8 times greater Ni concentration in the cathode bulk. It has previously been shown that Mn has a more significant impact on side reactions in the SEI than other transition metals and therefore may be chiefly implicated in reducing the cycle life here.^23^ It is also possible that differences in depth into the SEI where the transition metals are deposited might affect the ratios in the spectra, with Mn species potentially covered more quickly by the degraded electrolyte than Ni species. Accompanying the increase in Ni concentration at the graphite surface with cycling, Ni 2p spectra reveal changes in the chemical state of the Ni with cycle number, suggesting either a change of oxidation state or chemical surrounding as the cycling proceeds.

Interestingly, the Co 3p intensities remain very low, indicating that Co makes only a very minor contribution to the transition metals incorporated into the SEI, which may reflect the limited dissolution of Co from the cathode. This agrees well with literature reports for NMC111 that Co is less prone to dissolution than Mn and Ni.^83^ We note, however, that some modest Co incorporation into the SEI after 120 cycles is confirmed by the NEXAFS spectra of Figure 11.

**Figure 9:**
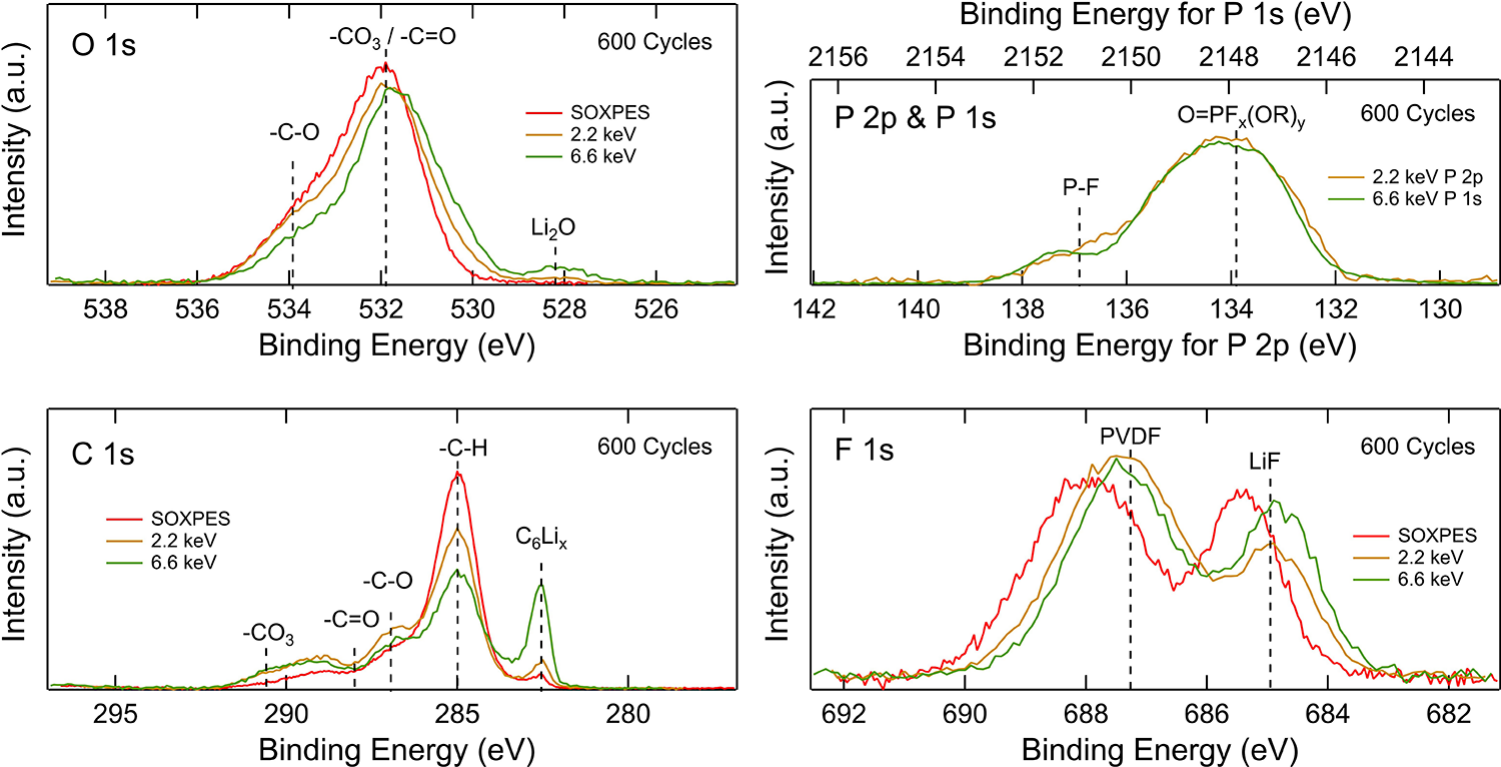
O 1s, P 2p, P 1s, C 1s, and F 1s core-level spectra of graphite electrodes cycled for 600 cycles obtained with excitation energies of 2.2 keV (yellow), 6.6 keV (green), and photoelectron kinetic energies of ∼315 eV (red).

Spectra were also acquired using different excitation energies to obtain information about differences in the chemistry throughout the SEI layer. Figure 9 shows the O 1s, P 2p, P 1s, C 1s and F 1s core-level spectra depending on excitation energy used, where the low-energy SOXPES data were measured at kinetic energies of ∼315 eV (excitation energies of 450–1000 eV) and the HAXPES data were measured at excitation energies of both 2.2 and 6.6 keV. The O 1s spectra show a higher relative intensity of the 528.2 eV binding energy peak with increasing excitation energies, indicating that the Li_2_O is mainly formed in the inner SEI layer. Conversely, the intensities at 533.9 eV decrease relative to the -CO_3_ peak, indicating that the −C–O species are mainly present in the outer SEI. This cannot be distinguished after 10 cycles but is seen for all samples cycled further, suggesting that this process is part of the SEI evolution (see Figure S8). In the F 1s spectra, the LiF peak at 684.9 eV is higher for the 6.6 keV excitation energy than the 2.2 keV excitation energy, indicating that the LiF is mainly present in the inner SEI layer. Interestingly, the SOXPES measurement shows an even higher intensity for the LiF, but this is likely due to beam damage after extended X-ray exposure. Given the greater propensity for beam damage at lower excitation energies, the SOXPES measurements were always performed last. A similar intensity distribution is also observed after 10, 120, and 300 cycles in Figure S9, indicating that no major change in depth distribution of the LiF takes place during the cycling. The C 1s spectra show that the hydrocarbon peak at 285.0 eV and the −C–O species at 286.8 eV are mainly present in the outer SEI and that the graphite peak is covered by SEI. Other carbon-based SEI species seem to be distributed throughout the layer as the intensity changes are small. The P 2p and P 1s spectra show that the peaks have similar intensities independent of excitation energy, suggesting that P-containing species are present throughout the SEI. Note that the P 1s spectrum has been overlaid on the P 2p spectrum.

**Figure 10:**
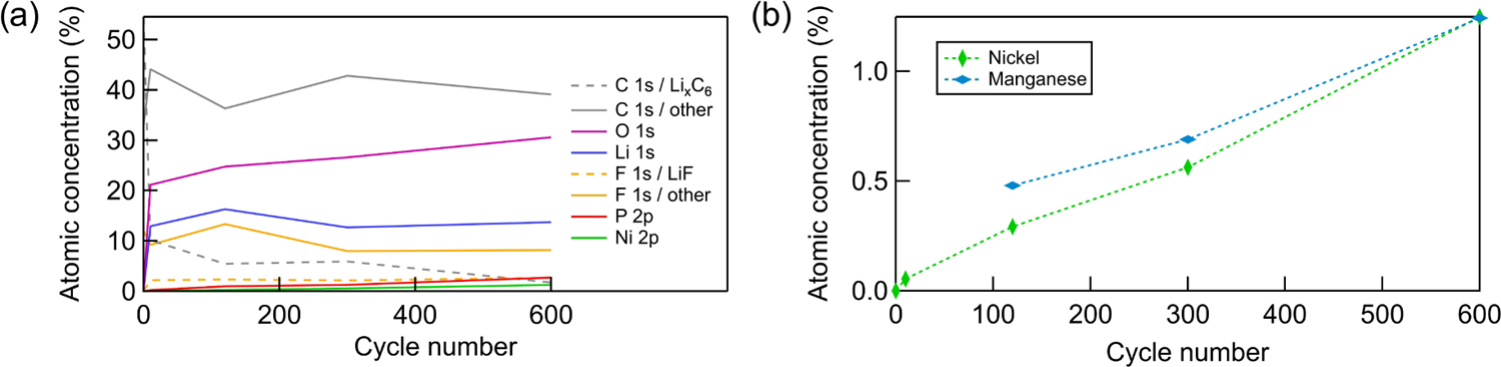
Elemental quantification of the surface of graphite electrodes. (a) Quantification of the main elements based on the survey spectra obtained at an excitation energy of 2.2 keV, with the ratio between different carbon and fluorine components determined from C 1s and F 1s spectra obtained at the same excitation energy. (b) Comparison of Ni and Mn concentrations based on the cross section-corrected intensity ratio between the 3p peaks.

Figure 10 displays the calculated atomic concentrations for the surfaces of the different graphite electrodes. The C_6_Li*_x_* concentration rapidly decreases with cycle number due to coverage of the graphite by the SEI layer. During the first cycles, a large amount of O-, C- and Li-containing species are deposited corresponding to SEI formation. The Li concentration remains almost constant during the following cycles, whereas the O concentration continues to slowly increase, likely due to the continuing decomposition of electrolyte solvent. The P concentration gradually increases with increasing cycle number, supporting the changes in the XPS data showing continued LiPF_6_ decomposition. Also, the Ni concentration increases with increasing cycle number, indicating that transition-metal incorporation into the SEI proceeds during prolonged cycling. The evolution of the Ni and Mn concentrations based on the intensity of their 3p core-level regions is shown in Figure 10b, revealing higher Mn concentrations at low cycle numbers. The relative Ni contribution seems to gradually increase with cycle number such that the concentrations become similar after 600 cycles, showing that the rate of transition-metal incorporation into the SEI changes for the different metals.

**Figure 11:**
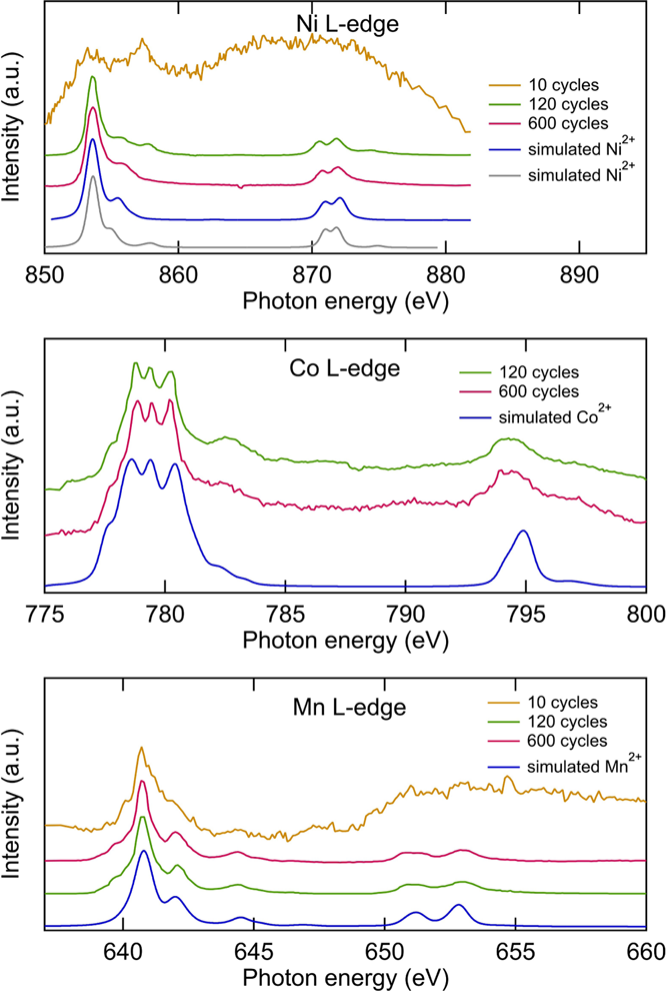
Ni, Co, and Mn L-edge NEXAFS spectra of graphite electrodes after different numbers of cycles, and simulated spectra obtained using CTM4XAS (see Table 1).

To further understand the role played by transition metals in the SEI layer, L-edge NEXAFS measurements were performed (see Figure 11). The Ni L_3_ edge after 600 cycles exhibits a larger peak at 853.6 eV, which primarily corresponds to Ni^2+^ and a smaller peak at 855.8 eV that can arise from both Ni^2+^ and Ni^3+^ contributions.^84^ The ratio between these peaks thus provides a useful indication of the oxidation state, which after 600 cycles is mainly Ni^2+^ as the contribution from the peak at 855.6 eV is relatively low. That Ni is present in its 2+ oxidation state is also confirmed by the close fit between the simulated Ni^2+^ spectra and the experimental data, agreeing with what has previously been observed on graphite.^23^ The ratio of the integrated L_3_ intensity to the total L-edge intensity is helpful in determining the spin state of the Ni,^60,85^ with a ratio between 0.72 and 0.76 indicating high spin and between 0.63 and 0.70 indicating low spin.^60^ For both the electrodes cycled at 120 and 600, the ratio was 0.75, which is well within the range for high spin. The high spin configuration is also supported by the well-resolved multiplet structure, i.e., a sharp shoulder peak on the high energy side of the 853.6 eV peak and a peak splitting of the L_2_ peak.^60^ Some differences are, however, apparent, with a satellite peak at 857.7 eV present after 120 cycles.^86^ This is captured in the simulation by changing the charge-transfer parameters, i.e., this indicates a different hybridization of the Ni ions with the surrounding species, possibly related to a change of ligands.^86^ The relative intensity of this peak is even larger after just 10 cycles, consistent with the SEI layer surrounding the Ni ions gradually changing during cycling. Alternatively, the surroundings of the Ni ions may depend on whether they are deposited in the inner or outer SEI layer. Further interpretation is challenging due to the low intensity and thus low signal-to-noise ratio of the Ni L-edge after 10 cycles. Nevertheless, this change in the chemical surrounding of the probed Ni ions is also supported by the HAXPES data in Figure 8b.

The Co L-edge shows small intensities after 120 and 600 cycles, making the peaks clearly discernible. This indicates primarily the presence of Co^2+^ based on comparison with experimental data for other Co^2+^compounds from the literature^87−89^ and with a Co^2+^ spectrum calculated using CTM4XAS. This is also consistent with other studies that observe Co in its divalent state on graphite electrodes.^23,90^ Similarly, for the Mn, the oxidation state appears to be predominantly Mn^2+^ based on comparison with reference and calculated spectra.^62,63^ Mn^2+^ has also been detected on negative electrodes in previous reports,^23,90^ where an ion-exchange reaction between Mn and Li ions was suggested.^91^ It was later suggested that the Mn^2+^ ions are instead transported through the electrolyte as neutral complexes, which become chemisorbed on the graphite after diffusing through the outer SEI layer to the inner part.^92^ In this case, Mn is found to be reduced during lithiation of the graphite and reoxidized during subsequent delithiation.^92^ Recently, it has been suggested that the Mn can react with the SEI components, making it revert to its oxidized state.^23^ This may explain why Mn is mainly detected in its divalent state herein, as the electrodes are measured in their discharged state when the graphite has been delithiated and the Mn has had enough time to react.

## Conclusions

In summary, the main source of capacity loss in NMC811–graphite cells during the first 200 cycles is found to be slippage due to electrolyte reduction at the graphite electrode. With further cycling, active material loss from the cathode becomes more significant, as does impedance growth. From a combination of SOXPES, HAXPES, and NEXAFS, we resolve the changes in interfacial chemistry occurring at both anode and cathode for these different degradation regimes. The CEI layer formed on the NMC cathode during the initial cycles is mainly composed of organic, oxygen-rich species on the outer surface, with more inorganic compounds closer to the electrode. Similarly, the outer part of the SEI layer formed on the graphite anode is rich in organic species, while the inner part shows large contributions from inorganic salts. Both the CEI and SEI are found to increase in thickness with cycling; however, the SEI is initially thicker and thickens more rapidly than the CEI over the course of aging, consistent with the direction of slippage observed.

During the first 200 cycles, Mn at the NMC surface becomes gradually more reduced, while the Co at the surface shows little change. Li_2_CO_3_ impurities present on the pristine NMC are found to gradually decompose over several hundred cycles, corresponding with the gradual increase in the NMC cutoff voltage related to slippage. Li_2_CO_3_ decomposition appears to correlate with increasing LiF content in the CEI, which is expected to contribute to increased cell impedance with the ongoing cycling, highlighting the importance of an inert (CO_2_- and moisture-free) storage environment for Ni-rich NMC electrodes. Parallel processes such as LiPF_6_ decomposition may also be involved in the LiF formation. Changes in the NMC are accompanied by the incorporation of Mn and Ni into the graphite SEI, indicating transition-metal dissolution, migration, and subsequent deposition. Interestingly, transition-metal incorporation does not follow the NMC stoichiometry, as has previously been assumed. Mn is initially much more prevalent while the ratio of Ni increases with cycle number, as the SEI continues to thicken. The chemical environment of the incorporated Ni is also seen to change, indicating changes in the surrounding ligands that may relate to ongoing reactions involved in slippage.

From 300 cycles onwards, the Mn, Ni, and Co at the NMC surface are found to be significantly more reduced and little Li_2_CO_3_ remains. The reduced transition metals are likely a result of the significant structural transformation of the NMC surface to a spinel/rock-salt layer, which is expected to contribute to the concurrent increase in impedance. This is accompanied by further transition-metal incorporation at the anode, with the relative proportion of Ni increasing with cycle number and Co becoming detectable, although still in much smaller quantities. We see a general alignment between the reduction of certain transition metals at the cathode and increasing incorporation of the same elements at the anode. This closely corresponds with the thickening of the CEI, spinel/rock-salt layer, and SEI, with all of these processes continuing (or even accelerating) after 300 cycles.

We rationalize these findings on the basis that slippage occurring during the first 200 cycles, which is primarily responsible for capacity fade during this period, eventually pushes the upper cutoff voltage of the cathode into a regime where the transition metals undergo significant surface reduction and dissolution. This leads to increased transition-metal incorporation into and thickening of the anode SEI, contributing to further slippage and thus exacerbating cathode degradation. The increase in cell impedance accompanying the growth of these surface layers reduces the accessible capacity and contributes to the ongoing capacity fade observed. We thereby show how the interplay between the surface degradation reactions occurring at the cathode and anode results in an accelerated capacity fade, understanding that we expect to inform the design of mitigation strategies to improve cycle life.

## Supporting Information Available

Potential profiles and DVA plots of NMC811–graphite cells, explanation of electrode slippage, and additional XPS spectra of NMC811 and graphite electrodes (PDF)
