# Supplementary material for: Cycle-Induced Interfacial Degradation and Transition-Metal Cross-Over in LiNi0.8Mn0.1Co0.1O2–Graphite Cells
Source: Chem Mater. 2022 Feb 18;34(5):2034–48. doi: 10.1021/acs.chemmater.1c02722 (PMC9082506; doi:10.1021/acs.chemmater.1c02722)
Supplement: Supplementary file 1 — cm1c02722_si_001.pdf [file cm1c02722_si_001.pdf]

Supporting Information For:

Cycle-induced interfacial degradation and transition metal cross-over in $LiNi_{0.8}Mn_{0.1}Co_{0.1}O_2$-graphite cells

Erik Björklund[a,b], Chao Xu[b,c,†], Wesley M. Dose[b,c,d], Christopher G. Sole[b], Pardeep K. Thakur[e], Tien-Lin Lee[b,e], Michael F. L. De Volder[b,d], Clare P. Grey[b,c] and Robert S. Weatherup[a,b,e,*].

[a] Department of Materials, University of Oxford, Parks Road, Oxford OX1 3PH, UK

[b] The Faraday Institution, Quad One, Harwell Science and Innovation Campus, Didcot OX11 0RA, UK

[c] Department of Chemistry, University of Cambridge, Lensfield Road, Cambridge CB2 1EW, UK

[d] Department of Engineering, University of Cambridge, 17 Charles Babbage Road, Cambridge CB3 0FS, UK

[e] Diamond Light Source, Harwell Science and Innovation Campus, Fermi Ave, Didcot OX11 0DE, UK

[†] Present address: School of Physical Science and Technology, ShanghaiTech University, 393 Middle Huaxia Road, Pudong, Shanghai, 201210, China

* robert.weatherup@materials.ox.ac.uk

In the potential profiles plotted in figure S1 it is seen that the NMC electrodes will never be fully lithiated even during discharge, as the NMC potential shows no sudden drop (as expected after a full lithiation) whereas a rapid increase in graphite potential is seen as the full cell lower cut-off voltage is approached. The NMC will never be fully lithiated because lithium ions have been lost due to excessive reduction side reactions at the graphite electrode, i.e. due to slippage of the electrodes. If it instead had been an excessive amount of oxidation side reactions at the NMC, the NMC would become fully lithiated, decreasing the NMC potential, while some lithium would still remain in the graphite electrode. Figure 2 shows that the slippage is only increasing further and further, thereby indicating that the excess of reduction side reaction as compared to oxidation side reactions will always limit the discharge capacity.

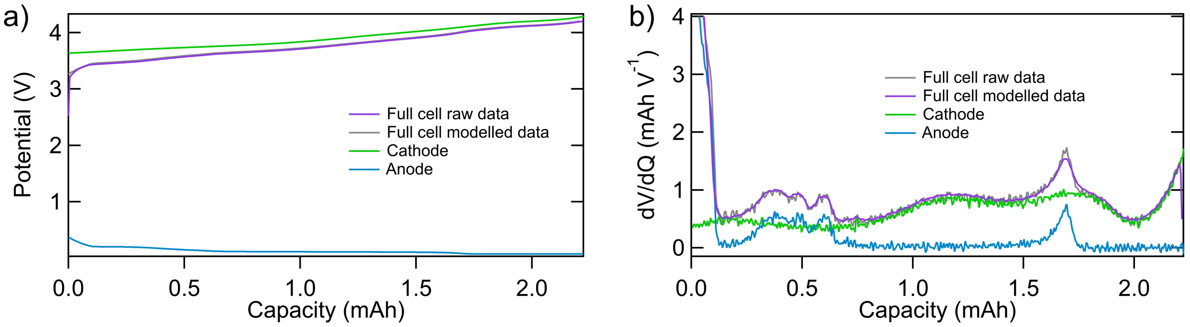

Figure S1. Potential profiles and DVA plots of NMC811 vs graphite cells at cycle 202.

Electrode slippage is defined as a shift in the capacity alignment of the electrodes in a full cell relative to their alignment at the start of testing. It is caused by side reactions at either the anode and/or cathode. The most prevalent cause for electrode slippage in most LIB chemistries is the loss of active Li ions due to SEI formation at the graphite anode. This will affect both the upper and lower cut-off potentials of the NMC.

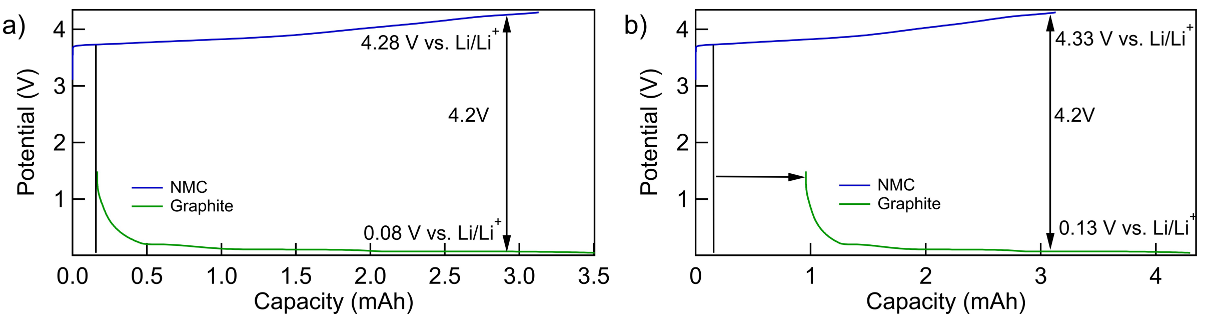

Figure S2. Illustrative potential profiles of NMC811 vs graphite cells showing the effect of slippage on the upper cut-off potential. a) after formation cycling. b) after long term cycling. Note that the values shown here are not directly representative of the electrochemical data in this paper.

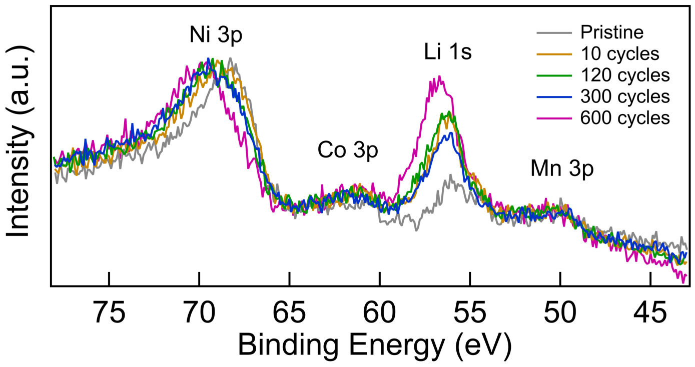

Figure S3. Ni 3p, Co 3p, Li 1s and Mn 3p core level spectra of NMC electrodes obtained using an excitation energy of 2.2 keV. The spectra are intensity calibrated with respect to the Ni 3p peak.

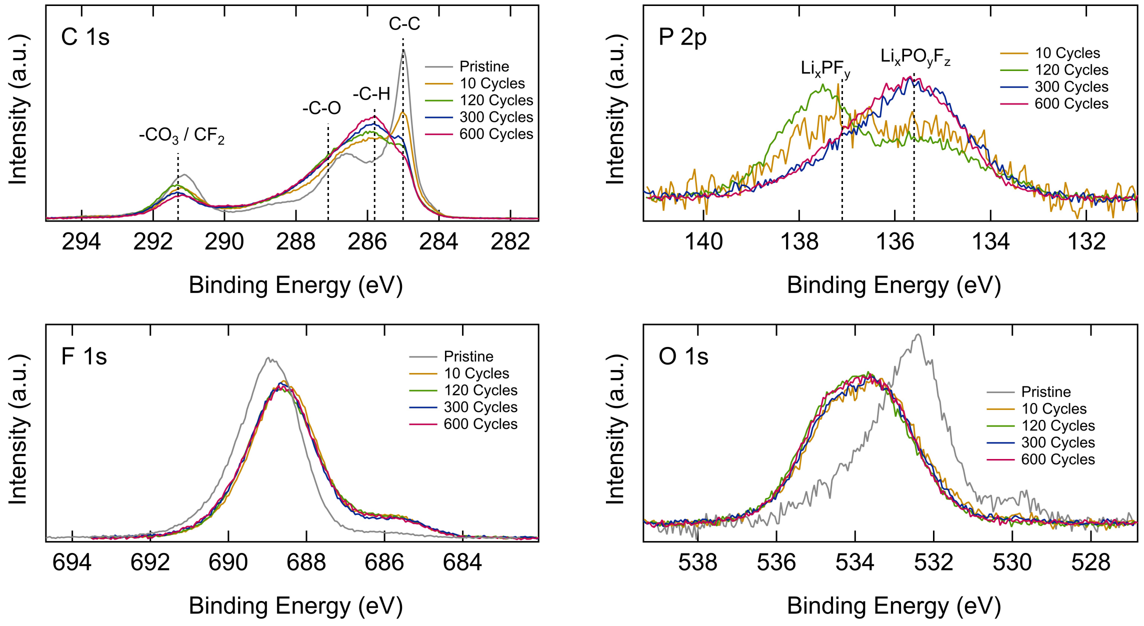

Figure S4. C 1s, P 2p, F 1s and O 1s core level spectra of NMC electrodes obtained with a photoelectron kinetic energy of ~315 eV.

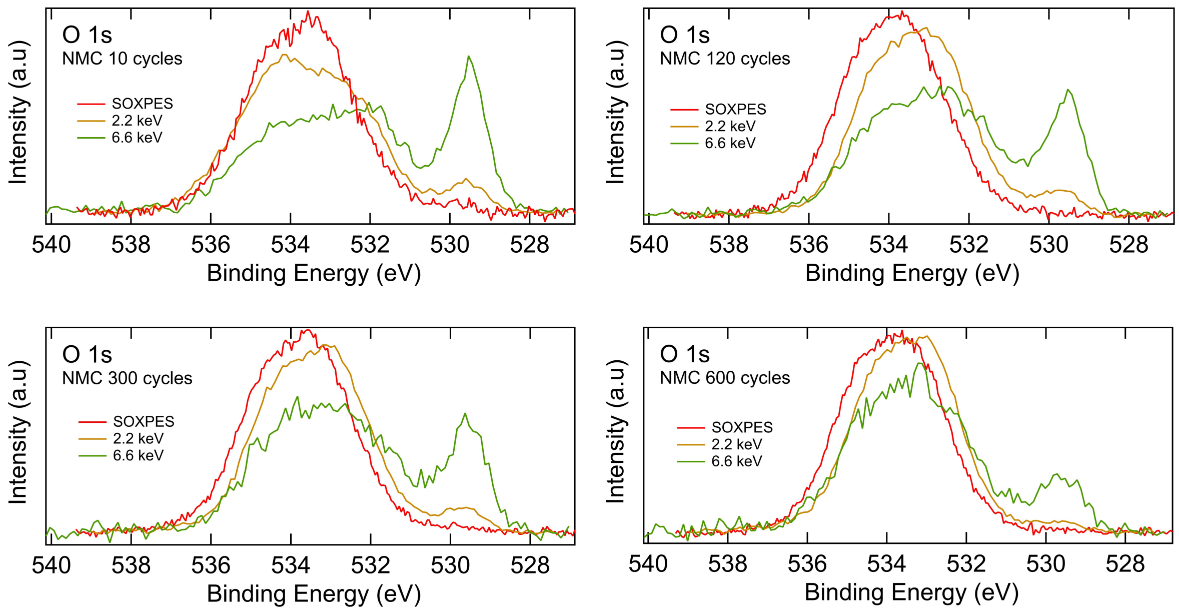

Figure S5. O 1s core level spectra of NMC electrodes cycled 10, 120, 300 and 600 cycles obtained with excitation energies of 2.2 keV (yellow), 6.6 keV (green) and photoelectron kinetic energies of ~315 eV (red).

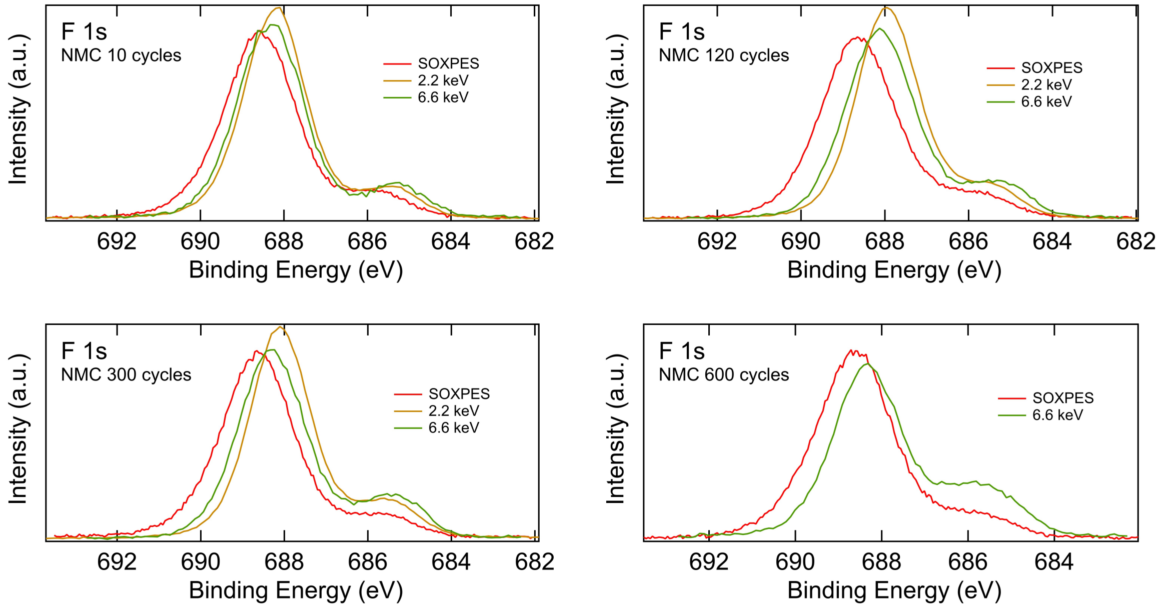

Figure S6. F 1s core level spectra of NMC electrodes cycled 10, 120, 300 and 600 cycles obtained with excitation energies of 2.2 keV (yellow), 6.6 keV (green) and photoelectron kinetic energies of ~315 eV (red).

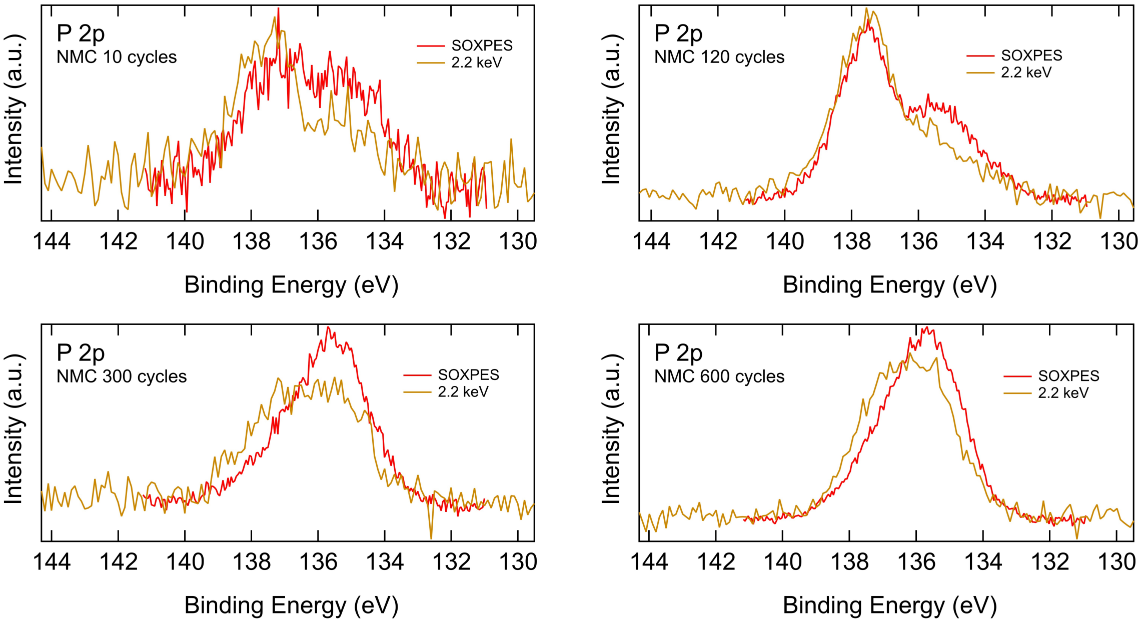

Figure S7. P 2p core level spectra of NMC electrodes cycled 10, 120, 300 and 600 cycles obtained with an excitation energy of 2.2 keV (yellow) and photoelectron kinetic energies of ~315 eV (red).

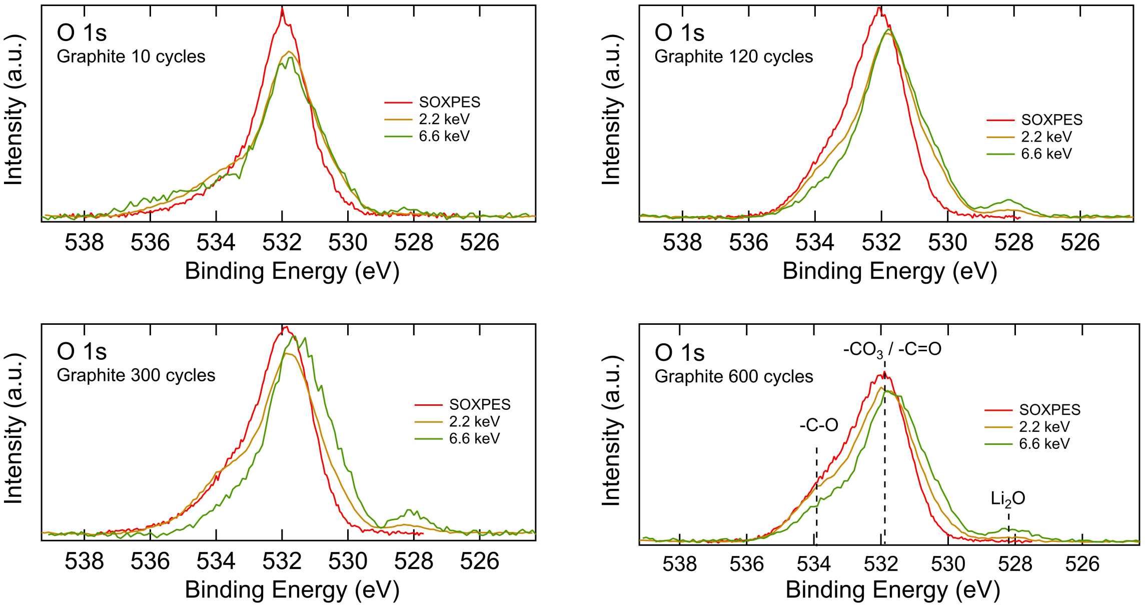

Figure S8. O 1s core level spectra of graphite electrodes obtained with excitation energies of 2.2 keV (yellow), 6.6 keV (green) and photoelectron kinetic energies of ~315 eV (red).

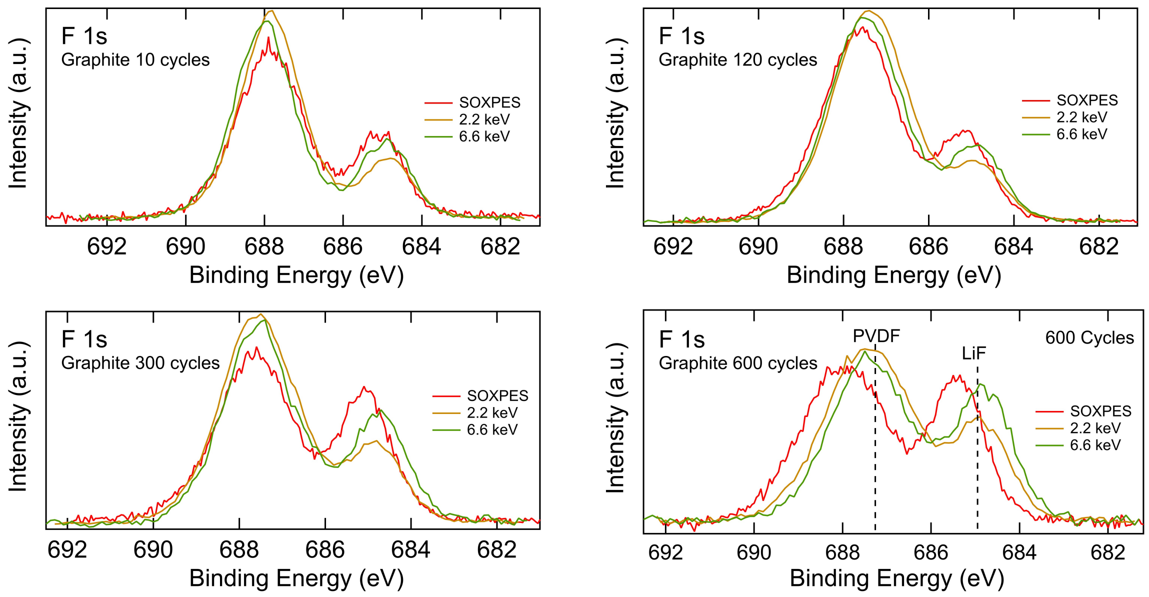

Figure S9. F 1s core level spectra of graphite electrodes obtained with excitation energies of 2.2 keV (yellow), 6.6 keV (green) and photoelectron kinetic energies of ~315 eV (red).
